# Supplementary material for: Whistler echo trains triggered by energetic winter lightning
Source: Nat Commun. 2024 Aug 21;15:7166. doi: 10.1038/s41467-024-51684-0 (PMC11339424; doi:10.1038/s41467-024-51684-0)
Supplement: Supplementary file 1 — Supplementary Information [file 41467_2024_51684_MOESM1_ESM.pdf]

Supplementary information

Whistler echo trains triggered by energetic winter lightning

I. Kolmašová[1,2*†], O. Santolík[1,2†], and J. Manninen[3]

[1] Department of Space Physics, Institute of Atmospheric Physics of the Czech Academy of Sciences, Prague, Czechia

[2] Faculty of Mathematics and Physics, Charles University, Prague, Czechia

[3]Sodankylä Geophysical Observatory, Sodankylä, Finland

* Corresponding author. Email: iko@ufa.cas.cz

†These authors contributed equally to this work.

Content:

Supplementary figure S1: **Lightning maps from January, 3, 2017**

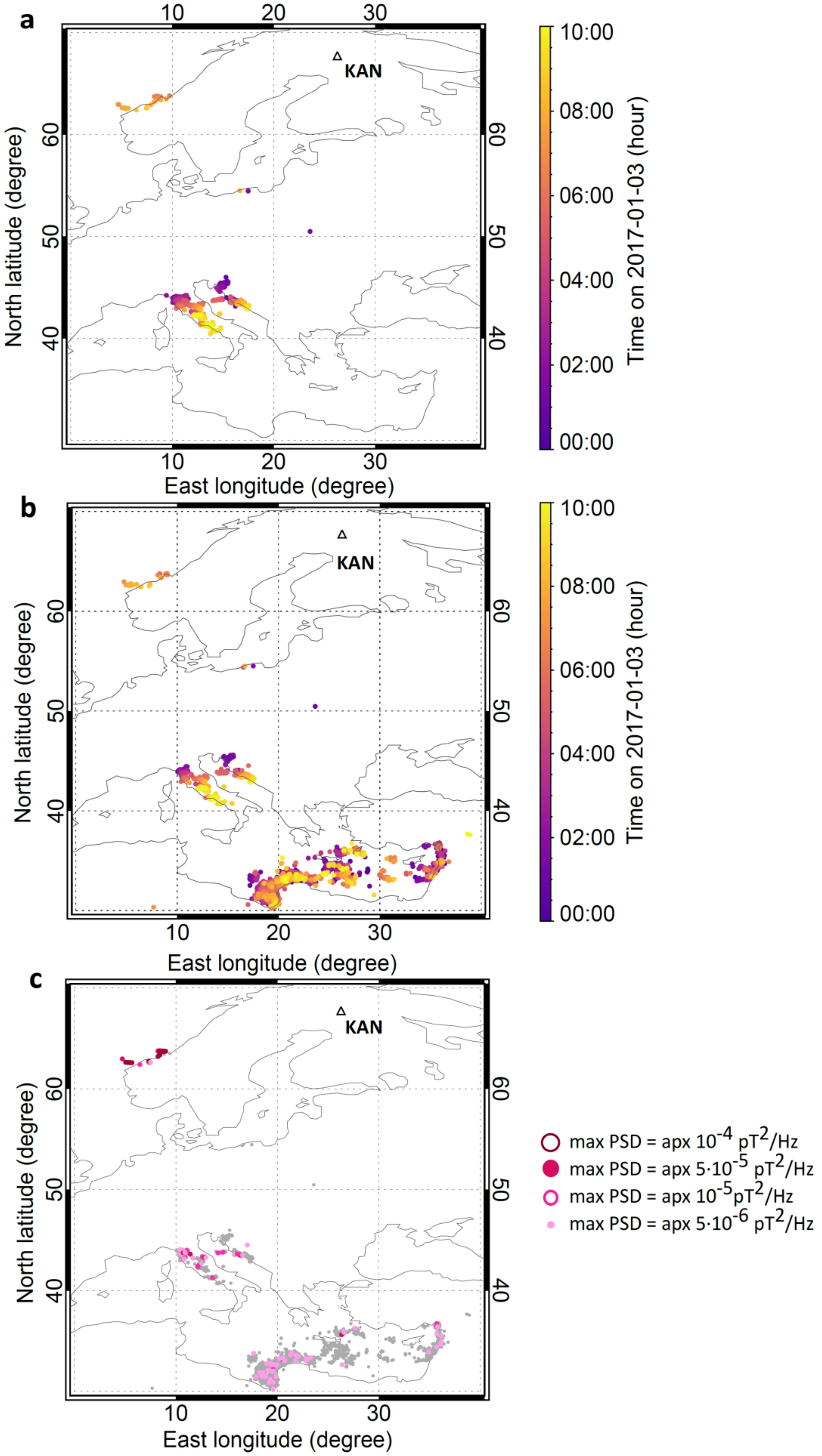
a
b
c
KAN
10
20
30
40
50
60
East longitude (degree)
North latitude (degree)
10:00
08:00
06:00
04:00
02:00
00:00
Time on 2017-01-03 (hour)
max PSD = apx 10^-4 pT^2/Hz
max PSD = apx 5·10^-5 pT^2/Hz
max PSD = apx 10^-5 pT^2/Hz
max PSD = apx 5·10^-6 pT^2/Hz

Supplementary Figure S1. **Lightning maps from January, 3, 2017**. **a** Locations of lightning discharges detected by EUCLID. **b** Locations of lightning discharges detected by WWLLN. The colors correspond to the time of lightning occurrence. **c** The locations of lightning discharges from the consolidated list (in grey) are shown, with the source lightning discharges for echo trains highlighted by pink-purple color coding based on the maximum power spectral density of the magnetic field of the first echo whistler. Source data are provided as a Source Data file. The maps were plotted using the IDL® procedures (a product of Exelis Visual Information Solutions, Inc., a subsidiary of Harris Corporation).
